# Acceptance and commitment therapy for fatigue interference in advanced gastrointestinal cancer and caregiver burden: protocol of a pilot randomized controlled trial

**DOI:** 10.1186/s40814-021-00837-9

**Published:** 2021-04-20

**Authors:** Catherine E. Mosher, Ekin Secinti, Kurt Kroenke, Paul R. Helft, Anita A. Turk, Patrick J. Loehrer, Amikar Sehdev, Ahmad A. Al-Hader, Victoria L. Champion, Shelley A. Johns

**Affiliations:** 1 Department of Psychology, Indiana University-Purdue University Indianapolis, 402 North Blackford Street, LD 124, Indianapolis, IN 46202 USA; 2 Indiana University School of Medicine, Center for Health Services Research, Regenstrief Institute, 1101 W. 10th Street, Indianapolis, IN 46202 USA; 3 Indiana University School of Medicine, Indiana University Melvin and Bren Simon Comprehensive Cancer Center, Indiana Cancer Pavilion, 535 Barnhill Drive, Suite 473, Indianapolis, IN 46202 USA; 4 Indiana University School of Nursing, 1111 Middle Drive, NU 340G, Indianapolis, IN 46202 USA

**Keywords:** Acceptance and commitment therapy, Fatigue, Symptom management, Metastatic gastrointestinal cancer, Family caregiver burden, Randomized controlled trial

## Abstract

**Background:**

Fatigue interference with activities, mood, and cognition is one of the most prevalent and bothersome concerns of advanced gastrointestinal (GI) cancer patients. As fatigue interferes with patient functioning, family caregivers often report feeling burdened by increasing responsibilities. Evidence-based interventions jointly addressing cancer patient fatigue interference and caregiver burden are lacking. In pilot studies, acceptance and commitment therapy (ACT) has shown promise for addressing symptom-related suffering in cancer patients. The current pilot trial seeks to test a novel, dyadic ACT intervention for both advanced GI cancer patients with moderate-to-severe fatigue interference and their family caregivers with significant caregiving burden or distress.

**Methods:**

A minimum of 40 patient-caregiver dyads will be randomly assigned to either the ACT intervention or an education/support control condition. Dyads in both conditions attend six weekly 50-min telephone sessions. Outcomes are assessed at baseline as well as 2 weeks and 3 months post-intervention. We will evaluate the feasibility, acceptability, and preliminary efficacy of ACT for improving patient fatigue interference and caregiver burden. Secondary outcomes include patient sleep interference and patient and caregiver engagement in daily activities, psychological flexibility, and quality of life. We will also explore the effects of ACT on patient and caregiver physical and mental health service use.

**Discussion:**

Findings will inform a large-scale trial of intervention efficacy. Results will also lay the groundwork for further novel applications of ACT to symptom interference with functioning and caregiver burden in advanced cancer.

**Trial Registration:**

ClinicalTrials.gov, NCT04010227. Registered 8 July 2019.

**Supplementary Information:**

The online version contains supplementary material available at 10.1186/s40814-021-00837-9.

## Background

Gastrointestinal (GI) cancers (e.g., colorectal, esophageal, liver, pancreatic, stomach cancer) are among the most common cancers in the USA, and the majority are discovered at advanced stages [1]. Fatigue is a highly prevalent symptom, with up to 68% of advanced colorectal cancer patients reporting moderate-to-severe fatigue [2, 3]. Among GI cancer patients, fatigue often co-occurs with a number of symptoms and substantially impacts daily activities and quality of life (QoL) [3–7]. Among advanced cancer patients, fatigue and related symptoms have been linked to prolonged hospitalizations and readmissions [8].

As fatigue and related symptoms affect patient functioning, family caregivers cope with demanding role changes that may impact their mental and physical health [9–12]. Among caregivers of colorectal cancer patients, greater caregiving burden (i.e., impact of caregiving on various aspects of their lives) has been associated with impaired QoL [13]. This burden includes extensive time spent performing caregiving duties to the neglect of other vital activities, such as self-care [13–16].

Evidence-based interventions reducing both patient fatigue and caregiver burden in GI and other advanced cancers are lacking. Cochrane meta-analyses of pharmacologic and behavioral interventions for fatigue in advanced cancer patients characterized the evidence as inconclusive [17, 18]. Additionally, behavioral interventions for cancer caregivers have produced small to medium effects on caregiving burden and QoL [19–21]. Most of these interventions were delivered to patient-caregiver dyads coping with early-stage cancer and did not have a symptom or distress criterion for study entry [19–21]. Thus, there is a significant need to develop interventions for symptomatic, advanced GI cancer patients and caregivers.

One behavioral intervention that shows potential for reducing symptom-related suffering in cancer and improving QoL is acceptance and commitment therapy (ACT) [22–25]. Rather than focusing on symptom reduction, the goal of ACT is to increase psychological flexibility so that unwanted symptoms, feelings, and thoughts interfere less with meaningful activities [26, 27]. Psychological flexibility is defined as fully experiencing the present moment while persisting in actions aligned with personal values [27]. In a pilot randomized controlled trial (RCT) in metastatic breast cancer, telephone-based ACT showed strong evidence of feasibility and promise for reducing fatigue interference with functioning compared to education/support [28]. ACT has also been found to reduce distress in various adult populations [29–31], although it has rarely been applied to caregivers of adults [32, 33].

The primary aim of this NIH-funded pilot RCT is to evaluate the feasibility and acceptability of delivering telephone-based ACT to advanced GI cancer patients and their caregivers. Our second aim is to test the potential effects of ACT on patient fatigue interference and caregiver burden (primary outcomes) as well as patient sleep interference and patient and caregiver engagement in daily activities, psychological flexibility, and QoL (secondary outcomes). We hypothesize that ACT will lead to improved outcomes as compared to education/support. This control condition involves supportive listening and education on medical center and community resources, consistent with common interventions in clinical settings. Our third aim is to explore the potential effects of ACT on patient and caregiver physical and mental health service use (tertiary outcomes). The current paper presents the rationale, design, methods, and analytic plan for this RCT.

## Methods/design

### Overview of study design

**Fig. 1:**
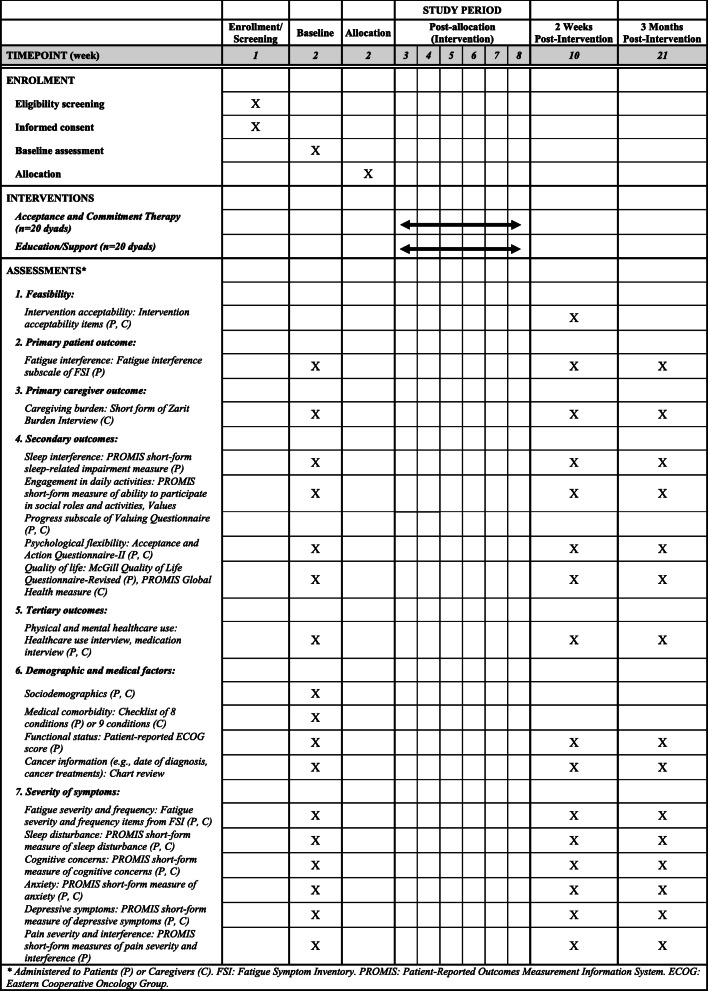
Schedule of enrollment, interventions, and assessments (Standard Protocol Items: Recommendations for Interventional Trials)

This pilot RCT examines the feasibility and acceptability of delivering telephone-based ACT to advanced GI cancer patient-caregiver dyads. Participants are recruited from the Indiana University Melvin and Bren Simon Comprehensive Cancer Center (IUSCCC) and Eskenazi Health in Indianapolis, Indiana. Figure 1 provides the enrollment, intervention, and assessment schedule. A minimum of 40 dyads will be randomized in equal numbers to six weekly 50-min telephone sessions of ACT or six weekly 50-min telephone sessions of education/support. Outcomes are assessed at baseline, 2 weeks post-intervention (primary endpoint), and 3 months post-intervention. ACT participants also complete qualitative interviews on ACT’s acceptability. This trial was registered in ClinicalTrials.gov (NCT04010227) and approved by the IU Institutional Review Board. Any study amendments will be approved by the Institutional Review Board prior to implementation and will be reported to the trial registry. This protocol paper is prepared according to Standard Protocol Items: Recommendations for Interventional Trials (SPIRIT) guidelines (see Additional file 1: SPIRIT checklist).

### Patient and caregiver eligibility criteria

Patient eligibility criteria are as follows: (a) ≥ 21 years old (required by NIH program announcement); (b) at least 3 weeks post-diagnosis of unresectable stage III or stage IV GI cancer (i.e., anal, colon, esophageal, gallbladder, liver, pancreatic, rectal, small intestine, or stomach cancer) as ascertained through medical records and consultation with the attending oncologist; (c) has an eligible, consenting family caregiver; (d) no significant cognitive impairment as ascertained by medical record review and a mental status questionnaire [34]; (e) English fluency; (f) working phone service; (g) in bed or chair less than half the day (patient-reported Eastern Cooperative Oncology Group [ECOG] score ≤ 2) [35]; (h) not enrolled in hospice; and (i) moderate-to-severe fatigue interference (mean score ≥ 2.5 on the Fatigue Interference subscale of the Fatigue Symptom Inventory [FSI]) [36, 37]. The clinical validity of this cut point is supported by our pilot research with advanced cancer patients. Caregiver eligibility criteria are as follows: (a) ≥ 18 years of age, (b) family caregiver who either lives with the patient or has visited them at least twice a week for the past month, (c) English fluency, (d) working phone service, and (e) significant caregiving burden (score ≥ 6 on the 6-item Zarit Burden Interview) [38] or distress on the 4-item Patient-Reported Outcomes Measurement Information System (PROMIS) anxiety or depression measures (*T*-score ≥ 60 [at least one standard deviation above the population mean] for anxiety or depression) [39]. The clinical cutoff for the Zarit Burden Interview has been established in advanced cancer caregivers [38]. We administer anxiety and depression measures at screening in order to enroll caregivers with high distress who may not endorse caregiving burden due to cultural factors.

### Recruitment

Initial patient eligibility is determined via electronic health record review and consultation with the attending oncologist. Prior to the COVID-19 pandemic, trained research assistants (RAs) approached patients and caregivers during oncology clinic visits or via phone. After COVID-19 restrictions were implemented, potential participants have only been approached through recruitment mailings and phone calls. The mailings include a consent form and letter of invitation with a phone number for opting out of further contact. An RA calls all patients who do not opt out approximately 1 to 2 weeks after the letter is mailed. The RA reviews the consent form in detail and answers questions. Interested patients identify a family caregiver and are screened for eligibility. Eligible and interested patients provide consent for study participation over the phone under a waiver of documentation of written informed consent. Caregivers of consenting patients receive a consent form via email or postal mail followed by telephone calls for eligibility screening and consent. With the patient’s permission, up to three of their family caregivers are consecutively screened for eligibility. If none of the caregivers are eligible and consent to participate, then the patient is ineligible for study participation.

### Randomization and blinding

Following baseline assessments, dyads are randomly assigned in equal numbers to ACT or education/support using stratified block randomization to balance the groups by performance status (patient-reported ECOG scores 0 or 1 vs. 2) [35]. Performance status informs treatment decisions in advanced GI cancer, such as whether to provide chemotherapy [40]. A study statistician used the R package “blockrand” [41, 42] to generate randomly varying block sizes of 2, 4, 6, and 8. Following randomization, study condition assignments are identifiable to participants, interventionists, and individuals mailing intervention materials. Other investigators, outcome assessors, and data analysts are blinded throughout the trial. Strategies to reduce the potential for unblinding include storing study condition assignments in separate data sheets that are only available to unblinded individuals. Additionally, the RA asks participants to refrain from discussing the intervention during follow-up assessments.

### Intervention

#### Acceptance and commitment therapy

**Fig. 2:**
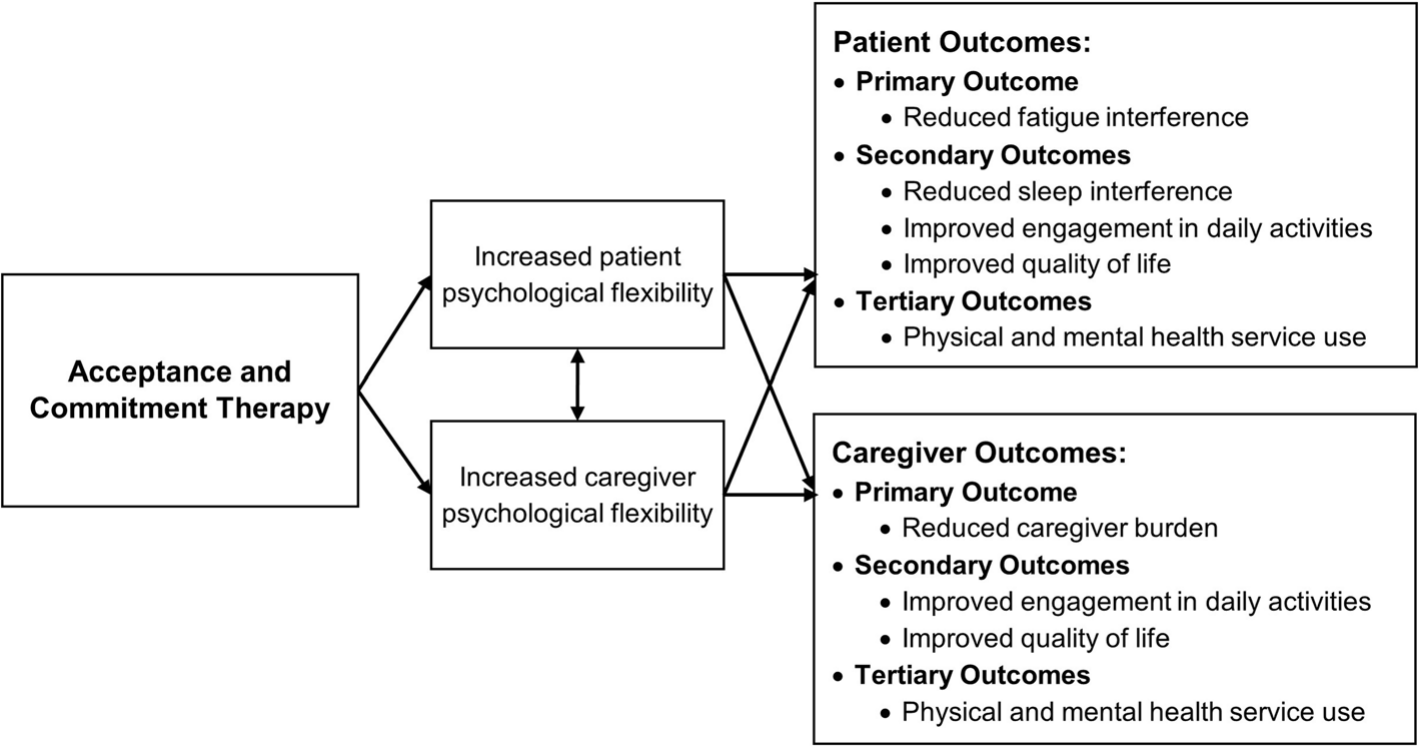
Conceptual model. Although psychological flexibility is a secondary outcome rather than a mediator in this pilot trial, our analyses will set the stage for formal mediation analyses in a future large-scale trial

We developed the ACT manual, which was informed by literature on the experiences of advanced GI cancer patients and caregivers [2, 3, 14, 43, 44], the ACT model [22, 27], previous ACT trials with cancer patients and other medical populations [25, 26, 28, 29, 31, 45, 46], and our clinical experience. A summary of intervention components is found in Table 1. Grounded in the ACT model [22, 27], the intervention is designed to reduce patient fatigue interference and caregiver burden by increasing psychological flexibility (see Fig. 2: conceptual model). Psychological flexibility is expected to increase through practicing mindfulness (e.g., meditations that encourage non-judgmental awareness of thoughts, feelings, and bodily sensations in the present moment), learning adaptive coping skills (e.g., acceptance, cognitive defusion, perspective-taking), identifying personal values (e.g., being a loving partner), and setting specific goals aligned with these values. Value-based action goals are in the SMART format (specific, measurable, achievable, relevant, and time-bound).

**Table 1:** Summary of core components of each intervention condition

Acceptance and commitment therapy	Education/support
•Discuss current attempts to control fatigue (if patient) or difficult thoughts and feelings (if caregiver) and their impact on quality of life •Practice mindfulness with the therapist during sessions and at home (e.g., awareness of the breath, body scan, leaves on a stream) •Practice cognitive defusion—noticing symptoms, thoughts, and feelings without being overwhelmed by them (e.g., passengers on the bus metaphor) •Observe and detach from difficult internal experiences (e.g., fatigue, thoughts, feelings) to cultivate a transcendent sense of self from which to notice and accept changing experiences •Identify important values (e.g., being a loving and engaged parent, serving one’s community) •Goal setting and practice of value-based actions	•Orientation to the medical center and treatment team; provide overview of quality-of-life concerns and discuss physical quality of life and symptoms •Discuss cancer-related social challenges (e.g., talking with children about cancer, employment concerns); tips on managing the household when ill; describe resources for addressing social challenges •Discuss common emotional reactions to cancer, including anxiety, depression, and stress, and cancer-related cognitive changes and describe available mental health services •Review common financial challenges related to cancer and its treatment and describe resources for addressing these challenges •Discuss methods of evaluating health information on the Internet and other modalities •Review previous session topics and refer to websites with cancer information

Patients and caregivers complete sessions 1 and 4–6 together via speakerphone. Sessions 2 and 3 are delivered to patients and caregivers separately. Holding both dyadic and individual sessions allows the therapist to meet the shared and unique needs of patients and caregivers. We adapted ACT to the dyad by incorporating joint mindfulness practices and leveraging the relationship during discussions. For example, participants discuss moments of emotional connection with each other as instances of mindfulness and provide encouragement to the other person during goal setting. Individual sessions with patients and caregivers include exploration of personal values. In addition, the workability of attempts to avoid fatigue (for patients) or unwanted thoughts and feelings about caregiving (for caregivers) is discussed. Thus, the focus of individual sessions is shifting from avoidant responses to the pursuit of value-based action despite fatigue or perceived caregiving burden.

During each session, patients complete three items from the FSI (i.e., average fatigue and fatigue interference with general activity and enjoyment of life) [36, 37], and both patients and caregivers complete two PROMIS anxiety items and two PROMIS depression items [39]. These reports allow the therapist to monitor participant fatigue and distress. Each participant’s home practice of mindfulness and other skills is also recorded, and practice for the week ahead is discussed. Each person receives handouts summarizing session topics and audio recordings that our team developed to guide mindfulness practices.

#### Education/support

Dyads in the education/support arm are directed to resources for practical and health information and contact information for psychosocial services. This condition controls for time and attention given to participants as well as therapist empathy, active listening, and education about resources. A similar comparator was used in our ACT pilot trial with metastatic breast cancer patients [28], and similar comparison groups have been used in studies with primarily advanced cancer patient-caregiver dyads [33, 47, 48]. The duration of sessions and number and order of dyadic vs. individual sessions are identical to those of the ACT condition. In addition, the same fatigue, depression, and anxiety assessments as those in the ACT condition are completed during each session [36, 37, 39]. A summary of the topics for each education/support session is found in Table 1. Education/support participants receive handouts on session topics and are asked to review them as homework. ACT concepts are not discussed.

#### Training of study staff and treatment fidelity

Training of interventionists and RAs involved didactics, live demonstrations, and role-plays. All assessments, interviews, and intervention sessions are digitally recorded. The principal investigator and trained doctoral students in clinical psychology are randomly reviewing 20% of assessments and qualitative interviews for adherence to the protocol.

Interventionists are doctoral level psychologists or master’s level mental health clinicians with extensive training and experience delivering ACT or supportive counseling. Each intervention is delivered by different therapists to prevent cross-contamination between study conditions. Interventionists received initial education in advanced GI cancer diagnosis and treatment, distress, and either ACT or supportive counseling techniques with training protocols developed in our team’s prior studies. Initial training also involved role-plays of sessions detailed in manuals. Licensed psychologists and doctoral students in clinical psychology review a random selection of recordings for adherence to the manual using checklists from our pilot research. All fidelity monitors have expertise in ACT or supportive counseling techniques and received training in the intervention protocol. Psychologists review sessions from both study conditions, whereas the doctoral students only review education/support sessions. During regular supervisory meetings with study therapists, psychologists provide treatment adherence scores (number of required topics and exercises covered in each session/total number of fidelity criteria) and discuss treatment fidelity issues.

### Retention

Several strategies are employed to enhance participant retention. All study staff, including RAs and therapists, have been instructed to clearly state expectations to participants and offer the option of completing follow-up assessments if they decide to discontinue the intervention. The use of the telephone for the entire study, including assessments, intervention sessions, and qualitative interviews, accommodates rural residents and those with low incomes. Participants are reminded of appointments via text, phone, or email. Finally, each person is mailed $40 in gift cards to a major retailor after completion of each assessment or interview.

### Adverse events and auditing

If any adverse events occur, such as increased risk of suicidality, a licensed mental health provider on the study team will refer the participant to mental health services. Adverse events will be reported to the Clinical Trial Management Center at IUSCCC comprised of experienced clinical researchers and clinicians and to the IU Institutional Review Board. All trials conducted at the IUSCCC are subject to quarterly audit by the Clinical Trial Management Center, which serves as an independent data monitoring committee.

### Study measures and data collection schedule

Figure 1 shows the study measures and the data collection schedule. Assessments are conducted by RAs blind to study condition. Patients complete a 30-min baseline assessment and two 25-min follow-up assessments. Caregivers complete a 25-min baseline assessment and two 20-min follow-up assessments. All measures have evidence of reliability and validity. Within approximately 2 weeks of the first follow-up, ACT participants are invited to complete a separate 30-min qualitative interview about ACT’s acceptability. Qualitative interviews are conducted with patients and caregivers separately.

#### Feasibility and acceptability data

Feasibility is assessed by accrual rates, attrition, and adherence (i.e., session attendance in both study conditions, home practice completion for ACT participants). Reasons for non-adherence to the intervention and study withdrawal are recorded. At the first follow-up, acceptability of both study conditions is assessed with helpfulness ratings on a scale from 1 (did not help at all) to 5 (extremely helpful) for number and length of sessions, topics, therapist, and telephone format [49]. Participants also rate the extent to which the telephone sessions met their expectations on a scale from 1 (not at all) to 5 (extremely). The RA is blind to study condition, as the questions apply to either condition. For ACT participants, acceptability is also assessed via qualitative interviews on the perceived helpfulness and impact of ACT components (see Additional file 2 for interview protocol).

#### Primary patient-centered outcomes

The primary outcome measure for patients is the 7-item Fatigue Interference subscale of the FSI [36, 37]. Items assess the extent to which fatigue in the past week interfered with activities, such as bathing, dressing, and housework, ability to concentrate, enjoyment of life, and mood on 11-point scales (0 = no interference; 10 = extreme interference). The primary outcome measure for caregivers is the 12-item short form of the Zarit Burden Interview [38, 50], which evaluates personal strain and role strain due to caregiving on 5-point scales (0 = never; 4 = nearly always).

#### Secondary outcomes

(1) Patient *sleep interference* is assessed using the 8-item PROMIS sleep-related impairment measure, which evaluates perceived interference of sleep problems with activities, mood, and cognition [51, 52]. (2) Patient and caregiver *engagement in daily activities* is assessed with the 6-item PROMIS measure of ability to participate in social roles and activities [53]. The items, which are reverse-coded, measure difficulty engaging in social and recreational activities as well as usual work (including housework). Patients and caregivers also report their progress in living consistently with personal values by completing the 5-item Value Progress subscale of the Valuing Questionnaire [54]. (3) Patient and caregiver *psychological flexibility* is evaluated with the 7-item Acceptance and Action Questionnaire-II (AAQ-II) [55]. (4) Patient *QoL* is assessed with the 15-item McGill Quality of Life Questionnaire-Revised, which was designed for patients with life-threatening illnesses and evaluates physical, existential, and social well-being [56]. Caregiver *QoL* is assessed with the 10-item PROMIS measure of global health, including physical, mental, and social well-being [57].

#### Tertiary outcomes

Patients and caregivers report their physical and mental healthcare use in five domains (e.g., ER visits, outpatient visits) in the past 3 months at baseline and over the study period [58, 59]. These reports of healthcare use were sensitive to change in a cancer trial [60]. At all time points, participants also report whether professionals referred them to support services and whether referred services were received [61, 62]. Additionally, at all time points, participants report current medications using established methods from prior trials [63, 64]. We will compute the total number of medications at each time point, as this variable had strong predictive validity for both healthcare use and costs in older adults over a 1-year period [65].

#### Descriptive variables

Patient and caregiver demographics and medical factors are assessed to characterize the sample. Caregivers also report their relationship to the patient. Patients and caregivers complete a checklist of eight or nine chronic health conditions, respectively [59]. Patient functional status is also assessed with the 1-item Patient Generated Subjective Global Assessment (PG-SGA), a patient-reported version of the ECOG score [35]. Patient cancer information, such as time since diagnosis and treatments, is collected via chart review. Finally, the severity of patient and caregiver symptoms is assessed. For both patients and caregivers, fatigue severity and frequency are measured with six items from the FSI [36, 37], and sleep disturbance, cognitive concerns, anxiety, and depressive symptoms are each assessed with a 6-item PROMIS measure [39, 51, 52, 66–68]. Patient pain is evaluated with 3-item and 4-item PROMIS measures of severity and interference, respectively [66, 69].

### Statistical analyses

#### Data management

Paper copies of assessments and other study materials are stored in locked filing cabinets in locked offices. Electronic data are kept in password-protected files on the secure, HIPAA-compliant IU server. Only IRB-approved members of the research team have access to the data. Participant identifiers are stored separately from participant data in a locked office or secure electronic files. Data are coded by participant identification number. Data are entered into the secure study database in REDCap and checked by two RAs with the supervision of the principal investigator. The principal investigator and data analysts will have access to the cleaned data set.

#### Preliminary analyses

All data will be assessed for missingness and multiple imputation with 50 imputed samples will be used [70]. We will also compare participants and those who decline participation or withdraw on demographic and medical factors using t-tests and Chi-square analyses.

We will assess for differences in baseline factors (e.g., demographic, medical and healthcare use variables) between study conditions [71]. Any differences will be considered when interpreting findings, and factors that vary between the groups will be included as covariates in sensitivity analyses.

#### Analysis for aim 1

Our analysis plan for aim 1 focuses on the feasibility and acceptability of the ACT intervention. Feasibility will be assessed by accrual rates, attrition, and adherence. We will judge this trial as feasible if (1) at least 60% of screened eligible dyads enroll in the study [72] and (2) at least 70% of randomized dyads complete 5–6 intervention sessions and the 2-week follow-up. These benchmarks for feasibility are based on our own pilot work and other published trials with advanced cancer patients and caregivers [19, 20, 72]. Acceptability of ACT and education/support sessions will be descriptively assessed with ratings of the helpfulness of the number and length of the sessions, topics, therapist, and telephone format on a scale from 1 (did not help at all) to 5 (extremely helpful) [49]. We will judge this trial as acceptable if at least 70% of patients and caregivers rate ACT as moderately to extremely helpful (i.e., an average score ≥ 4 on 1 to 5 Likert scale items). We will also descriptively examine whether the ACT and education/support sessions met patients’ and caregivers’ expectations on a scale from 1 (not at all) to 5 (extremely). Additionally, we will conduct descriptive within-dyad comparisons of all acceptability ratings.

For ACT participants, acceptability will also be assessed with semi-structured qualitative interviews on the perceived helpfulness of intervention components and their impact on functioning and well-being. The interviews will be audio-recorded, transcribed verbatim, and analyzed using an immersion/crystallization approach [73]. A clinical psychologist and health communication specialist, along with the RA, will develop a coding system. The analysis will consist of two phases: open and focused coding [73, 74]. Open coding facilitates the development of a code list for further analysis. In this phase, the analysts will independently label each line of data to reflect meanings or themes emerging from the text. This is done iteratively, combining, adding, or eliminating themes, until analysts agree on a set of emergent thematic categories (codes). In focused coding, codes derived in open coding are independently applied to all transcripts. To ensure that analysts remain consistent with coding, every fourth transcript will be coded by all three analysts, who will meet to discuss and reach consensus on coding. Atlas-ti software will facilitate coding, and qualitative results will inform intervention refinement for the subsequent large-scale RCT.

#### Analysis for aim 2

Given that this is a pilot study, our data analytic approach will be to derive effect size estimates rather than test for statistical significance. Effect sizes will be cautiously interpreted, as they are likely to be biased and imprecise [75]. A linear mixed model repeated measures approach (SAS Proc-Mixed) will be used to examine ACT’s effects on primary and secondary outcomes. For outcome measures that only patients or caregivers complete (e.g., patient fatigue interference or caregiver burden), models will include main effects of time (as categorical) and study group and the time-by-study group interaction. Treatment effects will be evidenced by the interaction between time and study group (i.e., group mean differences after the intervention but no such differences at baseline). Models will include random intercepts to account for nonindependence of individuals’ scores across time. For outcomes reported by patients and caregivers (e.g., psychological flexibility), multilevel modeling for dyadic data will be used [76, 77]. Models will include the main effects of time, study group, and social role (patient vs. caregiver) as well as all two- and three-way interactions between these variables. The time × study group × role interaction will estimate the degree to which treatment effects are different for patients and caregivers. Dyadic models will include random intercepts for patients and caregivers, as well as the covariance between the intercepts to account for nonindependence across time and across partners.

We plan to recruit at least 40 advanced GI cancer patients and 40 caregivers, consistent with the recommendations of the Stage Model of Behavior Therapies Research for examining feasibility and effect sizes in a pilot trial [78]. Although our analyses focus on effect sizes rather than statistical significance, we calculated power for 34 patients and 34 caregivers at 2 weeks post-intervention (assuming a 15% attrition rate) [49]. For each of the primary outcomes (patient fatigue interference and caregiver burden), we will have 80% power (*p* = .05, two-tailed) to detect a large intervention effect (*d* = .99) in a linear mixed model [79]. We expect medium to large effects of ACT on primary and secondary outcomes based on the literature. For example, ACT had large effects on distress and QoL outcomes in late-stage ovarian cancer patients (*d*s = .89–1.69) compared to cognitive-behavioral therapy [25] and a medium effect (*d* = − .59) on fatigue interference compared to education/support among metastatic breast cancer patients with moderate-to-severe baseline fatigue interference in our pilot trial [28]. We do not have preliminary data to estimate an effect of ACT on cancer caregiver burden.

#### Analysis for aim 3

We will use logistic and Poisson regression models to explore the effects of ACT on patient and caregiver physical and mental health service use (e.g., number of patient ER visits, patient and caregiver use of counseling and/or psychiatric medication). Logistic and Poisson regression analyses are appropriate for binary and count outcome data, respectively. Analyses will examine study condition as a predictor of health service use over the entire study period, controlling for baseline service use. Support service referrals will be a covariate in analyses of mental health service use.

## Discussion

The present pilot trial addresses the top-rated concern of advanced GI cancer patients—fatigue’s interference with activities, mood, and cognition [80]—and family caregiver burden. Evidence-based interventions to address these concerns are lacking. The ACT model [27] and pilot studies of ACT with cancer patients [24–26, 28, 45, 46] provide a strong rationale for the current trial. This project will be a catalyst for addressing a critical gap in the evidence-based care of this large, understudied population.

ACT is hypothesized to reduce fatigue interference in cancer patients, as it emphasizes mindful acceptance of the present moment—including internal experiences such as fatigue—and engagement in activities aligned with personal values. Our pilot trial with metastatic breast cancer patients was the first to examine ACT’s effect on fatigue interference [28]. The intervention showed strong evidence of feasibility and promise regarding its impact on this outcome [28]. This study expands this line of research to both advanced GI cancer patients with significant fatigue interference and their caregivers. Of note, the use of a fatigue interference eligibility criterion for patients and a caregiver burden/distress criterion for caregivers allows us to focus on dyads with greater need for intervention, an approach rarely used in the broader psycho-oncology literature [19, 81].

The current ACT intervention is one of the first to use a dyadic approach [33]. Although preliminary evidence suggests that ACT improves QoL outcomes in patients with cancer and other chronic conditions [25, 26, 28, 29, 31, 45], these trials have almost exclusively focused on patients. Because caregivers often experience significant burden [13, 14], evidence-based interventions should be developed to improve their QoL and capacity to balance important roles and activities. A dyadic intervention may improve outcomes for both patients and caregivers as they reinforce each other’s practice of ACT skills. Additionally, the intervention will capitalize on the relational bond between family members. For example, when setting personal goals, each family member will describe the strengths and resources that the other person possesses to facilitate goal achievement. Thus, our dyadic ACT intervention aims to improve interdependent QoL and behavioral outcomes by leveraging the familial relationship.

Our ability to demonstrate the feasibility and promise of ACT will lead to a large-scale trial and ultimately fulfill an unmet need in the comprehensive care of advanced GI cancer patients and caregivers. Given widely available training in ACT, the intervention can be readily disseminated to clinicians. Results will also provide a foundation for a program of research focused on the novel application of ACT to symptom interference with functioning and caregiver burden in advanced cancer.

Results of this study will be published in a peer-reviewed journal and submitted to ClinicalTrials.gov within 12 months of the primary completion date. The trial protocol and data set will be available to qualified investigators within 6 months of the publication of the primary outcome paper. The investigators will be required to sign a data use agreement and obtain IRB approval before receiving the data set.

## Supplementary Information

**Additional file 1:.** SPIRIT checklist

**Additional file 2:.** Qualitative interview protocols for patients and caregivers

## Data Availability

Not applicable

## Abbreviations

AAQ-II: Acceptance and Action Questionnaire-II
ACT: Acceptance and commitment therapy
ECOG: Eastern Cooperative Oncology Group
FSI: Fatigue Symptom Inventory
GI: Gastrointestinal
PG-SGA: Patient Generated Subjective Global Assessment
PROMIS: Patient-Reported Outcomes Measurement Information System
QoL: Quality of life
RA: Research assistant
RCT: Randomized controlled trial
SMART: Specific, measurable, achievable, relevant, and time-bound
