# Supplementary material for: Acceptance and commitment therapy for fatigue interference in advanced gastrointestinal cancer and caregiver burden: protocol of a pilot randomized controlled trial
Source: Pilot Feasibility Stud. 2021 Apr 20;7:99. doi: 10.1186/s40814-021-00837-9 (PMC8056101; doi:10.1186/s40814-021-00837-9)
Supplement: Supplementary file 2 — Additional file 2:. Qualitative interview protocols for patients and caregivers [file 40814_2021_837_MOESM2_ESM.docx]

**Additional File 1**: Interview Protocol

Qualitative Interview Protocol for Patients

- First, I would like to get a sense of your overall experience with [therapist name]. In general, what did you think of the telephone program?
- Which parts of the program did you find to be most helpful?
- Not every part of the program may be helpful to every person, and we are looking for ways to improve the program. Which parts of the program did you find to be less helpful?
- Now remember when you did mindfulness practices with [therapist name] like picturing leaves on a stream or noticing your breath? What did you think of those?
  - Did you notice any changes as a result of the mindfulness practices?
    - Probes if yes: What changes did you notice? How is your daily life different as a result of these practices? Has it changed how you think about or deal with your fatigue and other symptoms?
- Did you have a chance to do any mindfulness practice this week?
- [If yes] What practice did you do? What changes, if any, did you experience after your practice?
- [If no] Was there a particular reason that you did not practice?
- Do you have any plans to practice mindfulness in the future?
- After you discussed your values with [therapist name], you were asked to set goals based on your values. Please give me an example of a goal that you set.
  - - What led you to set that goal?
    - How did it go for you?
    - How did it make you feel to [reflect back participant’s response]?
- Did you notice other changes in yourself as a result of participating in this program?
- Probes: What changes did you notice? Did it change how you behaved? Did it change how you thought or felt about your current situation, including your cancer and symptoms like fatigue?
- Did you notice any changes in [insert name of caregiver] as a result of participating in this program?
  - Probe: What changes did you notice? Did you notice changes in how they dealt with their current situation, including the realities of your cancer?
- Did you talk about the telephone program with [insert name of caregiver] outside of the sessions with [therapist name]?
  - [if yes] What did you talk about?
- Did you do any of the home practices with [insert name of caregiver]?
  - [if yes] Which practices did you do together?
- What did you think of the number of sessions? (i.e., were there too few, too many, was it about right?)
  - [if dissatisfied] How many sessions would you prefer?
- What did you think about doing two one-on-one phone sessions with [therapist name] and the rest of the sessions with [insert name of caregiver]?
  - [if dissatisfied] What would you have preferred? (Probe if unclear.)
- What did you think of how long the sessions lasted?
  - [if dissatisfied] What would have been a good length for the sessions?
- What did you think of doing the sessions over the phone?
  - Would you have preferred another form of communication besides the phone? (Probes: would you prefer in-person sessions, Internet-based sessions, or another format for this program?)
- What other feedback about the sessions with [therapist name] or the home practice do you want to share with me?
  - Would you add anything to the program?
  - Would you leave anything out of the program?
  - What else did you learn from the program?

Qualitative Interview Protocol for Caregivers

- First, I would like to get a sense of your overall experience with [therapist name]. In general, what did you think of the telephone program?
- Which parts of the program did you find to be most helpful?
- Not every part of the program may be helpful to every person, and we are looking for ways to improve the program. Which parts of the program did you find to be less helpful?
- Now remember when you did mindfulness practices with [therapist name] like picturing leaves on a stream or noticing your breath? What did you think of those?
  - Did you notice any changes as a result of the mindfulness practices?
    - Probes if yes: What changes did you notice? How is your daily life different as a result of these practices? Has it changed how you think about or deal with your current situation, including the realities of your spouse’s/family member’s cancer?
- Did you have a chance to do any mindfulness practice this week?
- [If yes] What practice did you do? What changes, if any, did you experience after your practice?
- [If no] Was there a particular reason that you did not practice?
- Do you have any plans to practice mindfulness in the future?
- After you discussed your values with [therapist name], you were asked to set goals based on your values. Please give me an example of a goal that you set.
  - - What led you to set that goal?
    - How did it go for you?
    - How did it make you feel to [reflect back participant’s response]?
- Did you notice other changes in yourself as a result of participating in this program?
- Probes: What changes did you notice? Did it change how you behaved? Did it change how you thought or felt about your current situation, including the realities of your spouse’s/family member’s cancer?
- Did you notice any changes in [insert name of patient] as a result of participating in this program?
  - Probe: What changes did you notice? Did you notice changes in how they dealt with their current situation, including their cancer and symptoms like fatigue?
- Did you talk about the telephone program with [insert name of patient] outside of the sessions with [therapist name]?
  - [if yes] What did you talk about?
- Did you do any of the home practices with [insert name of patient]?
  - [if yes] Which practices did you do together?
- What did you think of the number of sessions? (i.e., were there too few, too many, was it about right?)
  - [if dissatisfied] How many sessions would you prefer?
- What did you think about doing two one-on-one phone sessions with [therapist name] and the rest of the sessions with [insert name of patient]?
  - [if dissatisfied] What would you have preferred? (Probe if unclear.)
- What did you think of how long the sessions lasted?
  - [if dissatisfied] What would have been a good length for the sessions?
- What did you think of doing the sessions over the phone?
  - Would you have preferred another form of communication besides the phone? (Probes: would you prefer in-person sessions, Internet-based sessions, or another format for this program?)
- What other feedback about the sessions with [therapist name] or the home practice do you want to share with me?
  - Would you add anything to the program?
  - Would you leave anything out of the program?
  - What else did you learn from the program?
